# Xiyanping Plus Azithromycin Chemotherapy in Pediatric Patients with *Mycoplasma pneumoniae* Pneumonia: A Systematic Review and Meta-Analysis of Efficacy and Safety

**DOI:** 10.1155/2019/2346583

**Published:** 2019-08-29

**Authors:** Qiao Li, Zhi-Yong Li, Jie Zhang, Wen-Na Guo, Xiao-Meng Xu, Fa-Xin Sun, Hui Xu

**Affiliations:** School of Pharmacy, Collaborative Innovation Center of Advanced Drug Delivery System and Biotech Drugs in Universities of Shandong, Key Laboratory of Molecular Pharmacology and Drug Evaluation (Yantai University), Ministry of Education, Yantai University, Yantai, China

## Abstract

**Background:**

Xiyanping injection (XYP) is a well-known Chinese medicinal preparation reputed as a most effective alternative to antibiotics. XYP has been widely used in combination therapies to treat various infectious diseases, among which XYP plus azithromycin (AZM) chemotherapy is often used for the treatment of *Mycoplasma pneumoniae* pneumonia in pediatric patients (p-MPP) in China.

**Objective:**

The present study just aimed to confirm whether XYP can improve the clinical efficacy and safety of AZM chemotherapy for p-MPP by performing meta-analysis and systematic review.

**Methods:**

A meta-analysis was performed following the Preferred Reporting Items for Systematic Reviews and Meta-Analyses (PRISMA) guidelines. The randomized controlled trials (RCTs) concerning XYP plus AZM chemotherapy for p-MPP were selected, for which the main outcomes included overall response rate (ORR), antipyretic time, cough disappearance time, lung wet Rales disappearance time, hospitalization duration, and adverse drug reactions (ADRs). Based on the data extracted, the meta-analysis was conducted by using a standard data extraction form.

**Results:**

Nine RCTs involving 963 patients were included for meta-analysis. More concretely, the combination therapy showed the risk ratio (RR) and 95% confidence intervals (CI) of ORR and ADRs as (RR, 1.21 [95% CI, 1.15, 1.28]) and (RR, 0.37 [95% CI, 0.27, 0.51]), respectively. And other major outcomes were as follows: hospitalization durations (standard mean difference (SMD), −1.32 [95% CI, −1.48, −1.16]), antipyretic time (SMD, −1.26 [95% CI, −1.70, −0.83]), cough disappearance time (SMD, −1.07 [95% CI, −1.38, −0.75]), and the disappearance time of lung wet Rales (SMD, −0.83 [95% CI, −1.07, −0.60]). With statistically significant differences in various aspects, the combination therapy plus XYP displayed obvious advantages in contrast to AZM alone.

**Conclusion:**

Overall, XYP might reduce the incidence of ADRs and significantly improve the clinical efficacy for p-MPP receiving AZM chemotherapy.

## 1. Introduction

*Mycoplasma pneumoniae* pneumonia (MPP) is a kind of respiratory disease caused by mycoplasma infection, which has a high incidence and mortality and occurs more frequently in children [1, 2]. Macrolides have long been first choice for respiration infections, among which azithromycin (AZM) is widely used to treat *Mycoplasma pneumoniae* pneumonia in pediatric patients (p-MPP). However, the severe challenge of AZM chemotherapy is the high bacterial resistance rate, which could even be up to 90% in China [3]. In addition, there are obvious side effects involved such as gastrointestinal discomforts, allergies, immune disorders, hepatic injury, and the damage to the nervous system, which thus limit the clinical efficacy of AZM chemotherapy [4–7].

Traditional Chinese medicine (TCM) injections are a class of preparation with typical Chinese characteristics [8]. It has been used for the prevention, treatment, and cure of disorders or diseases for centuries [9]. With the increasing use of traditional medicine worldwide during the last few decades, the use of TCM injections in China has also been on the rise [10, 11]. Due to the advantage of both rapid effect and good safety, TCM injections have long been used especially in combination with conventional chemotherapies for the purpose of synergism and toxicity attenuation [12]. Xiyanping injection (XYP) is just a kind of TCM preparation made from andrographolide, the main active component of *Andrographis paniculata* (Burm. f.) Nees, a well-known medicinal herb with efficacy of cooling blood to dissipate stasis and clearing heat toxicity [13]. XYP displays potent antibacterial and antiviral effects but no detectable antibiotic resistance in clinical practice. Therefore, it is reputed as a most effective alternative to antibiotics and has been widely used in China by combination therapy to treat various infectious diseases, including bronchitis, tonsillitis, and bacillary dysentery [14, 15]. Several recent reports further demonstrated the clinical application of XYP plus AZM chemotherapy for the treatment of p-MPP [12, 16, 17].

So far there is no evidence-based support for the therapeutic superiority of XYP in combination with AZM chemotherapy in p-MPP. Furthermore, the optimal combination therapy regimen for desired efficacy and safety still remains unclear. Based on the data from related randomized controlled trials, we are here to systematically reevaluate all related studies to further confirm whether XYP plus AZM chemotherapy improve the efficacy and reveal its safety and combination with AZM chemotherapy for p-MPP.

## 2. Materials and Methods

This meta-analysis was implemented by following the preferred reporting items for systematic reviews and meta-analyses guidelines (PRISMA guidelines). Ethical approval was not required, as the materials were published studies.

### 2.1. Search Strategy

Two reviewers (Qiao Li and Zhi-Yong Li) independently retrieved all the clinical randomized controlled trials using XYP plus AZM chemotherapy for p-MPP in Chinese and English database. The search strategies were as follows: (XYP injection OR Xiyanping injection OR Xiyanping) AND (Azithromycin [Mesh] OR AZM) AND (*Mycoplasma pneumoniae* pneumonia OR Mycoplasma pneumonia OR MP OR MPP) AND (Children OR Pediatric). Chinese databases were identified as China National Knowledge Infrastructure Database (CNKI), Chinese Scientific Journals Full-Text Database (VIP), Wanfang Database, and China Biological Medicine Database (CBM), while the English databases were Medline, Embase, and PubMed. All retrievals were implemented using MeSH and free word, and the retrieval period lasted from the established time to August 2018. After rigorous evaluation of the similar or related SRs or meta-analysis, the trials meeting the inclusion criteria were finally selected from related references.

### 2.2. Inclusion Criteria

The criteria to include studies for further analysis were as follows: (i) the study design was a prospective, randomized controlled trial; (ii) it included patients with p-MPP; (iii) it randomized patients to a strategy of XYP plus AZM therapy and the parallel control group using AZM alone; and (iv) the outcome indicators measured for efficacy and safety evaluation included ORR, ADRs, hospitalization durations, antipyretic time, cough disappearance time, and lung wet Rales disappearance time.

### 2.3. Exclusion Criteria

The criteria to exclude studies from further analysis were as follows: (i) the study design did not have a clear standard of diagnosis and treatment; (ii) there were no the control groups or the control groups using interventions other than AZM in the study; (iii) literature on nonclinical studies such as reviews, pharmacological experiments, and animal experiments; (iv) clinical studies with unsatisfactory results of efficacy assessment or with unclear statistical analysis; and (v) clinical studies that did not explicitly describe randomized grouping methods. Their design was a prospective, randomized controlled trial.

### 2.4. Selection of Studies

Two reviewers (Qiao Li and Zhi-Yong Li) independently selected the studies meeting the predefined inclusion criteria. Any disagreements about selection were resolved by discussion between themselves or with third reviewer (Jie Zhang).

### 2.5. Data Extraction

Two reviewers (Qiao Li and Zhi-Yong Li) independently extracted and recorded all data in a standard extraction form. The collection data from each study were as follows: first author, year of publication, number of the case of trial and control, the age of the participants, male/female ratio, and the outcome indicator. Data on effectiveness, including ORR, hospitalization durations, antipyretic time, cough disappearance time, the disappearance time of lung wet Rales, and data on safety, including ADRs, were also extracted from the articles.

### 2.6. Outcomes

Our primary outcomes were measured for evaluation included: ORR, ADRs, and hospitalization durations. Our secondary outcomes were symptom-disappearing time, including antipyretic time, cough disappearance time, and the disappearance time of lung wet Rales.

### 2.7. Quality Assessment

We evaluated the risk of bias of included studies using the RCT bias risk assessment tool in Cochrane System Evaluator's Handbook version 5.1.0. Risk of bias assessment was utilized to address the following seven domains: random sequence generation, allocation concealment, blinding of participants and personnel, blinding of outcome assessment, incomplete outcome data, selective outcome reporting, and “other bias” [18]. For each RCT, the bias risk assessment was summarized to the following criteria: low risk, high risk, and unclear risk. Two researchers (Qiao Li and Wen-Na Guo) discussed the above criteria, when there were any disagreements, negotiating with the third reviewer (Jie Zhang), reaching consensus opinion.

### 2.8. Statistical Analysis

The interstudy heterogeneity among trials were assessed by using the *χ*^2^ and *I*^2^ tests. The *I*^2^ statistic approximates the proportion of total variation in the effect size estimates that is due to heterogeneity rather than the sampling error. *I*^2^ values above 25%, 50%, and 75% were taken as indicators of mild, modest, and high heterogeneity, respectively, and the differences were considered statistically significant with the *P* value lower than 0.10. The meta-analysis was implemented by using pooled effect estimates and fixed-effects and random-effects models of risk ratios (RR) and standard mean difference (SMD), calculated with 95% confidence intervals (CI). For all analyses, the results from the fixed-effects model are presented only when there was no heterogeneity between trials (*I*^2^ < 50%); otherwise, the results from the random-effects model are presented (*I*^2^ ≥ 50%). Sensitivity analyses were undertaken when high heterogeneity appeared (*I*^2^ ≥ 75%).

## 3. Results

### 3.1. Search Results

**Figure 1:**
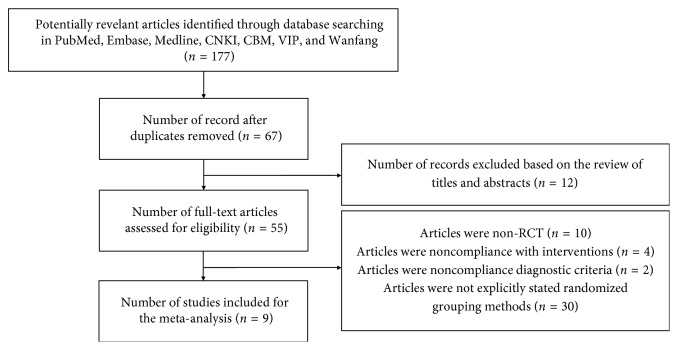
Flow chart depicting the number of studies included at each stage of the selection process. RCT, randomized controlled trial.

First of all, identification of eligibility of the studies was performed according to the technological process illustrated in Figure 1. By the present research strategy, 177 potentially relevant articles were found to meet the selection criteria, including 10 non-RCTs, 2 about interventions with noncompliance, 4 noncompliance with diagnostic criteria, and 30 not explicitly describing the randomized grouping methods. Finally, nine clinical trials were identified as prospective randomized controlled trials for further analysis, after 67 duplicates were removed and other 12 ones were excluded based on the review of titles and abstracts [16, 17, 19–25]. All the included nine trials randomized patients to either the treatment of AZM alone or XYP plus AZM chemotherapy, among which there were 7 studies utilizing the random number table method to divide the participants into the experiment or control group, one using the drawing lots and grouping method to allocate the subjects, and the other one adopting a randomly grouping method by a computer to divide participants into two groups. Moreover, none mentioned any withdrawal or dropout, and the subjects involved averagely aged 0.5 to 14 years, with no significant difference in gender ratio between the experimental and the control group (Table 1).

### 3.2. Quality Assessment of Included Studies

**Figure 2:**
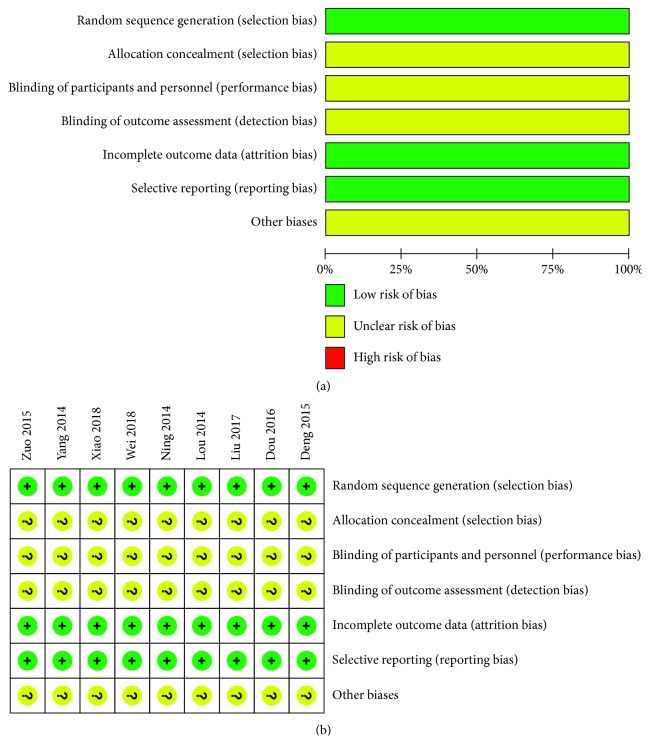
Graph (a) and summary (b) for risk of selection bias. The low risk and unclear risk of bias was illustrated by signs + and ?, respectively.

Quality assessment of the included studies was further performed according to risk of bias involved in meta-analysis. As illustrated in Figure 2, the bias might stem from selection, performance, detection, attrition, and reporting, which were possibly related to random sequence generation, allocation concealment, blinding of participants and personnel, blinding of outcome assessment, incomplete outcome data, or selective reporting. More concretely, there were no high risk of bias for all the included trials, along with low risk of bias found in random sequence generation, incomplete outcome data, and selective reporting. These findings clearly demonstrated a good reliability of the searching strategy and included criteria used for the present systematic review. In order to lower the selection bias, we indeed formulated clear and strict inclusion/exclusion criteria and performed analysis independently by two researchers (Qiao Li and Wen-Na Guo). When they presented inconsistent screening results, the publication would be subjected to an additional review by the third reviewer (Jie Zhang). Nevertheless, most of the included studies provide limited information for reliable risk judgment, thus leading to some unclear risks of bias, which were contributed to selection, performance, detection, and other unknown factors.

### 3.3. The Primary Outcomes

#### 3.3.1. Overall Response Rate

**Figure 3:**
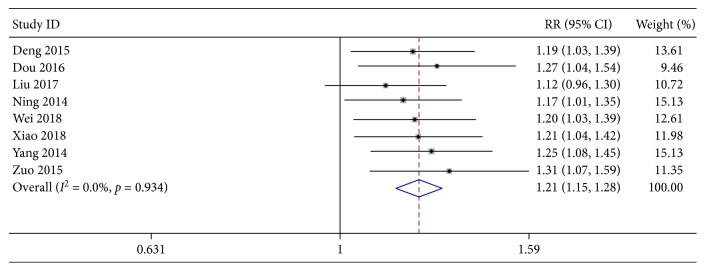
Forest plots for risk ratio (RR) of total effectiveness rate. Vertical line indicates “no difference” point between the two regimens; horizontal lines indicate the 95% confidence interval (CI). ♦, risk ratios; ◊, pooled risk ratios for all studies.

The over response rate (ORR) was evaluated according to the outcomes from 8 included RCTs. The test of interstudy heterogeneity among the trials showed *χ*^2^ = 2.41, *P*=0.934, and *I*^2^ = 0.0%, indicating that there was no significant heterogeneity among these included studies. Thus, the fixed-effects model was used for meta-analysis. As illustrated in Figure 3, the patients with XYP plus AZM chemotherapy showed significantly higher ORR values than those treated only with AZM (pooled RR 1.21, 95% CI [1.15, 1.28], *z* = 6.67, *P* < 0.001).

#### 3.3.2. Adverse Drug Reactions

**Figure 4:**
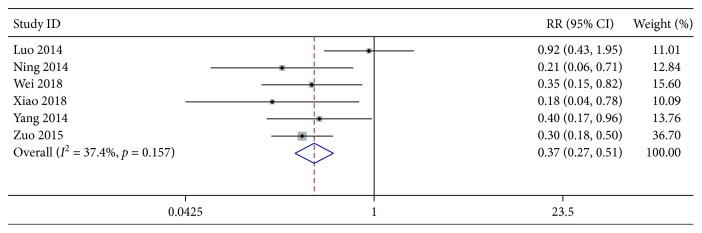
Forest plots for risk ratio (RR) of adverse reactions. Vertical line indicates “no difference” point between the two regimens; horizontal lines indicate the 95% confidence interval (CI). ♦, risk ratios; ◊, pooled risk ratios for all studies.

The information concerning with AZM-associated side effects such as rash, vomiting, and abdominal pain was extracted from 6 included studies. The test of interstudy heterogeneity showed no significant heterogeneity among the trials (*χ*^2^ = 7.99, *P*=0.157, *I*^2^ = 37.4%). As shown in Figure 4, the meta-analysis using fixed-effects model further revealed a significant difference in the incidence of AZM-related ADRs between AZM groups and those with XYP plus AZM chemotherapy (pooled RR 0.37, 95% CI [0.27, 0.51], *z* = 6.11, *P* < 0.001).

#### 3.3.3. Hospitalization Durations

**Figure 5:**
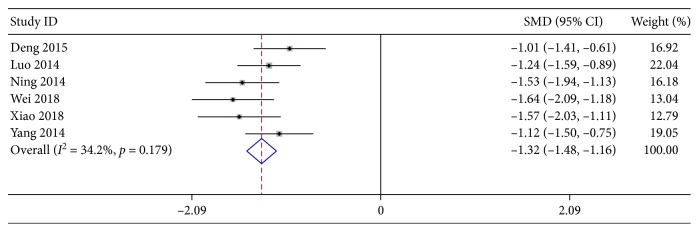
Forest plots for risk ratio (RR) of hospitalization durations. Vertical line indicates “no difference” point between the two regimens; horizontal lines indicate the 95% confidence interval (CI). ♦, risk ratios; ◊, pooled risk ratios for all studies.

Six articles were included to investigate how XYP plus AZM chemotherapy affected the hospitalization durations. The trials overall displayed no significant interstudy heterogeneity (*χ*^2^ = 7.60, *P*=0.179, and *I*^2^ = 34.2%); thus, the meta-analysis was performed by using the Mantel–Haenszel model. As shown in Figure 5, it was demonstrated that the patients with XYP plus AZM chemotherapy had significantly shorter hospitalization durations than those with AZM alone (pooled SMD −1.32, 95% CI [−1.48, −1.16], *z* = 15.74, *P* < 0.001).

### 3.4. The Secondary Outcomes

#### 3.4.1. Antipyretic Time

**Figure 6:**
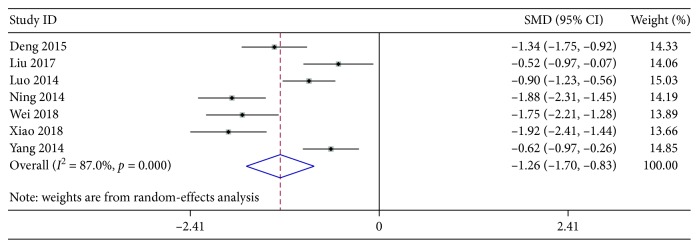
Forest plots for risk ratio (RR) of antipyretic time. Vertical line indicates “no difference” point between the two regimens; horizontal lines indicate the 95% confidence interval (CI). ♦, risk ratios; ◊, pooled risk ratios for all studies.

Seven studies with a total of 781 participants were included to investigate the difference in antipyretic time between AZM treatment groups and XYP plus AZM groups. Significant heterogeneity was found among the trials (*χ*^2^ = 46.03, *P* < 0.001, and *I*^2^ = 87.0%), and the evaluation by the random-effects analyzer revealed significant difference among these studies (pooled SMD −1.26, 95% CI [−1.70, −0.83], *z* = 5.72, *P* < 0.001) (Figure 6). As shown in Table 2, the sensitivity analysis further demonstrated that there was no significant change between the total effect amount and the combined effect amount after excluding some certain study, suggesting a stable result from the present meta-analysis.

#### 3.4.2. Cough Disappearance Time

**Figure 7:**
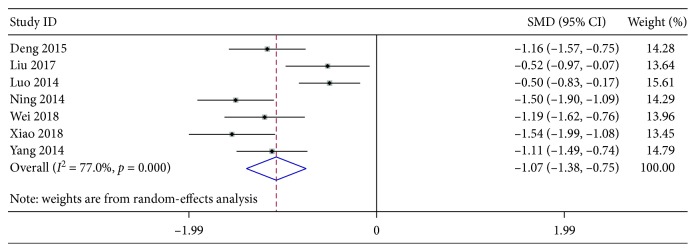
Forest plots for risk ratio (RR) of cough disappearance time. Vertical line indicates “no difference” point between the two regimens; horizontal lines indicate the 95% confidence interval (CI). ♦, risk ratios; ◊, pooled risk ratios for all studies.

There were a total of 7 studies involving 781 participants meeting the inclusion criteria. These studies overall showed a relatively high heterogeneity in this secondary outcome (*χ*^2^ = 26.11, *P* < 0.001, and *I*^2^ = 77.0%). In contrast to the treatment by AZM alone, the analysis based on the random-effects model clearly demonstrated the advantage of XYP plus AZM chemotherapy in shorting cough disappearance time with a statistically significant difference (pooled SMD −1.07, 95% CI [−1.38, −0.75], *z* = 6.58, *P* < 0.001) (Figure 7). In light of the *I*^2^ value more than 75%, sensitivity analysis was further performed. The result shown in Table 3 was similar to that of antipyretic time, also indicting some certain correlation between the two secondary outcomes of p-MPP.

#### 3.4.3. The Disappearance Time of Lung Wet Rales

**Figure 8:**
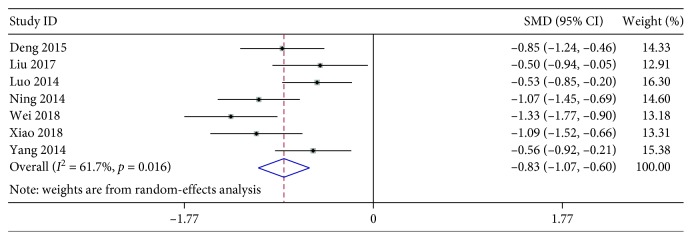
Forest plots for risk ratio (RR) of the disappearance time of lung wet Rales. Vertical line indicates “no difference” point between the two regimens; horizontal lines indicate the 95% confidence interval (CI). ♦, risk ratios; ◊, pooled risk ratios for all studies.

The heterogeneity test based on the 7 articles reporting relevant data showed that there was significant difference among these data (*χ*^2^ = 15.65, *P*=0.016, and *I*^2^ = 61.7%). Further evaluation by using a random-effects model revealed a pooled SMD of −0.83 with a 95% CI of −1.07 to −0.60. As illustrated in Figure 8, the findings indicated that XYP plus AZM chemotherapy is more likely to shorten lung wet Rales disappearance time than the treatment by AZM alone (*z* = 6.84, *P* < 0.001).

## 4. Discussion

Although the definite pathogenesis has not yet been fully elucidated, it has been demonstrated that the occurrence of MPP is closely associated with direct damage to lung from mycoplasma infection and endotoxin such as nutrition depletion, destruction of membrane fusion, invasive and inflammatory damage, and immune-mediated injuries [26]. Macrolide antibiotics are usually the first-line choice in clinic for infectious diseases, among which the antimicrobial agent azithromycin is the most commonly used one for the treatment of MPP [27]. However, long-term administration of AZM might lead to an increased risk of bacterial resistance, thus attenuating its therapeutic efficacy [28].

XYP is a kind of traditional Chinese medicine (TCM) preparation made from andrographolide, one of the major active components in the TCM herb *Andrographis paniculata* (Burm. f.) Nees that has long been used for cooling blood to dissipate stasis and clearing heat toxicity [29, 30]. A variety of studies have demonstrated extensive pharmacological activities of andrographolide, especially anti-inflammatory effect and antibacterial potency against most common Gram-positive and Gram-negative bacteria, such as *Staphylococcus aureus, Streptococcus*, *Escherichia coli*, *and pneumonia* [31], contributing to antibacterial and antiviral efficacy of XYP along with good safety and low rate of drug resistance [13]. Indeed this TCM preparation is reputed as an effective alternative to antibiotics and widely used in China for various infectious diseases, particularly by combination therapies with macrolide antibiotics [16, 17, 19–25].

By using systematic review and meta-analysis, our present work investigated the clinical efficacy and safety of XYP plus AZM chemotherapy for the treatment of MPP, a kind of respiratory disease common in pediatric population. Nine RCTs, which involved 963 pediatric patients with MPP were selected, including 559 males and 404 females aged 6 months to 14 years. All the experiment groups were treated with XYP plus AZM-based chemotherapy, whereas the control groups were treated only with AZM. The data were extracted for analysis according to evaluation of six major outcome indicators, such as ORR, ADRs, hospitalization durations, antipyretic time, cough disappearance time, and lung wet Rales disappearance time. Albeit moderate quality evidences, the findings clearly revealed that the clinical application of XYP plus AZM chemotherapy for p-MPP was associated with a significantly higher ORR than AZM alone. The analysis of outcomes further demonstrated statistical heterogeneity in ADRs, hospitalization durations, and the disappearance time of lung wet Rales. Meanwhile, both antipyretic time and cough disappearance time showed high heterogeneity among these studies. Taking into account the fact that the included studies did not include the basic information about the patients and the dosages, it is difficult to perform a subgroup analysis based on this. Thus, sensitivity analysis was performed to provide a counterbalance, and the results indicating that the major factors responsible for statistical heterogeneity in these outcomes might include XYP administration regime, the patients' condition, and potential involvement of other complications.

Nevertheless, there were still some limitations in this study. First of all, only the Chinese and English databases were retrieved in view of the fact that XYP is a kind of TCM preparation used in China. Resultantly, a limited number of trials carried out in China with insufficient sample sizes were included for the present systematic review and meta-analysis, which may reduce the test efficiency and cause some selection bias [12]. Furthermore, no included trial specifically described the blinding method and allocation concealment, thus tending to result in the bias in selectivity and implementation [12]. Meanwhile, most of the articles included in this study were related to positive results, which would likely lead to publication bias and the overestimation of actual treatment effects. All the abovementioned facts therefore indicated possible insufficient evaluations of these outcome indicators for p-MPP treatment.

## 5. Conclusion

In conclusion, the findings from the present meta-analysis and systematic review indicated that combination of XYP and AZM might be an effective and safe therapy for p-MPP in clinic. In order to get a more definite clinical conclusion for the efficacy and safety of XYP plus AZM chemotherapy, stricter and longer investigation should be performed on the basis of adequate data from multicenter RCTs in the future.

## Figures and Tables

**Table 1:** Characterization of enrolled trials and extracted data.

Study	Cases (T/C)	Age (years)	Male/female ratio	Outcome indicator
Deng and Li [16]	56/53	1–14	67/43	①②③④⑤
Dou et al. [19]	40/40	2–10	44/36	①
Liu et al. [20]	40/40	2–12	49/31	①②③④⑤
Luo [17]	75/75	0.5–13	85/65	②③④⑤⑥
Ning [21]	60/60	3.9–6.7	74/46	①②③④⑤⑥
Wei [22]	50/50	3–9	46/54	①②③④⑤⑥
Xiao [23]	48/48	1–12	63/33	①②③④⑤⑥
Yang [24]	63/63	2–13	73/53	①②③④⑤⑥
Zuo [25]	51/51	0.5–13	58/44	①⑥

Note: T, therapy group; C, control group; ①, overall response rate; ②, antipyretic time; ③, cough disappearance time; ④, the disappearance time of lung wet Rales; ⑤, hospitalization durations; ⑥, adverse drug reactions.

**Table 2:** Sensitivity analysis result of antipyretic time.

Study omitted	Estimate	[95% confidence interval]	
Deng and Li [16]	−1.25	−1.76	−0.74
Liu et al. [20]	−1.38	−1.83	−0.98
Luo [17]	−1.33	−1.84	−0.82
Ning [21]	−1.16	−1.60	−0.72
Wei [22]	−1.18	−1.65	−0.72
Xiao [23]	−1.16	−1.60	−0.71
Yang [24]	−1.37	−1.82	−0.92
Combined	−1.26	−1.70	−0.83

**Table 3:** Sensitivity analysis result of cough disappearance time.

Study omitted	Estimate	[95% confidence interval]	
Deng and Li [16]	−1.05	−1.42	−0.68
Liu et al. [20]	−1.15	−1.48	−0.82
Luo [17]	−1.17	−1.45	−0.89
Ning [21]	−0.99	−1.32	−0.66
Wei [22]	−1.05	−1.41	−0.68
Xiao [23]	−0.99	−1.32	−0.66
Yang [24]	−1.06	−1.44	−0.68
Combined	−1.07	−1.38	−0.75
